# Ferroptosis in epithelial ovarian cancer: a burgeoning target with extraordinary therapeutic potential

**DOI:** 10.1038/s41420-023-01721-6

**Published:** 2023-12-01

**Authors:** Danhua Ruan, Jirui Wen, Fei Fang, Yuqin Lei, Zhiwei Zhao, Yali Miao

**Affiliations:** 1 Department of Obstetrics and Gynecology, Key Laboratory of Birth Defects and Related Diseases of Women and Children of MOE, West China Second University Hospital, West China Campus, Sichuan University, Chengdu, 610041 Sichuan Province China; 2 State Key Laboratory of Biotherapy, West China Hospital, Sichuan University, Chengdu, 610041 Sichuan Province China; 3 Deep Underground Space Medical Center, West China Hospital, Sichuan University, Chengdu, 610041 Sichuan Province China; 4 West China School of Basic Medical Sciences & Forensic Medicine, Sichuan University, Chengdu, 610041 Sichuan Province China

**Keywords:** Ovarian cancer, Cell death

## Abstract

Epithelial ovarian cancer (EOC) is universally acknowledged as a terrifying women killer for its high mortality. Recent research advances support that ferroptosis, an emerging iron-dependent type of regulated cell death (RCD) triggered by the excessive accumulation of lipid peroxides probably possesses extraordinary therapeutic potential in EOC therapy. Herein, we firstly provide a very concise introduction of ferroptosis. Special emphasis will be put on the ferroptosis’s vital role in EOC, primarily covering its role in tumorigenesis and progression of EOC, the capability of reversing chemotherapy resistance, and the research and development of related therapeutic strategies. Furthermore, the construction of ferroptosis-related prognostic prediction systems, and mechanisms of ferroptosis resistance in EOC are also discussed. Finally, we propose and highlight several important yet unanswered problems and some future research directions in this field.

## Facts

- As one of the most lethal gynecologic malignancies, ovarian cancer is a terrifying women killer. And epithelial cancer accounts for 95% of ovarian cancer.
- Inconspicuous symptoms at early stage, high recurrence, and facilitated chemotherapy resistance have collectively made it an urgent necessity to define novel therapeutic targets and explore new effective therapeutic strategies for epithelial ovarian cancer.
- Ferroptosis, a distinct iron-dependent type of regulated cell death, has exhibited tremendous and excellent potential in the treatment of various cancers, including epithelial ovarian cancer.

## Open Questions

- Epithelial ovarian cancer is a disease of high heterogeneity on histopathological level. Does ferroptosis play a similar role and have the analogous therapeutic potential in all subtypes of epithelial ovarian cancer?
- How can we accurately examine and confirm the subtypes sensitive to ferroptosis-associated therapy in clinical practice?
- While emerging evidence has proved that a combination of ferroptosis-related theapy and ionization radiation, immunotherapy, or chemotherapy, can achieve a better anticancer effect, how can we effectively select the optimal combination for patients diagnosed at different stages of various subtypes?
- How to evaluate the accuracy of the ferroptosis-related prognosis-predictive models or systems under real clinical circumstances?
- What approaches can be adopted to overcome ferroptosis resistance in epithelial ovarian cancer therapy?

## Introduction

Ranking eighth around the globe [1], ovarian cancer (OC), one of the most fatal gynecological malignancies, has always been considered as an enormously terrifying women killer. According to the conservative estimation of American Cancer Society, only in USA, the somber numbers of new cases and deaths of OC will respectively reach up to 21410 and 13770 in 2022 [2]. As a highly heterogenous cancer, OC can be basically classified into a group of malignancies based on different cell/site of origin, histopathologic staging, risk factors, treatment, and prognosis [3–5]. Thereinto, nonepithelial ovarian cancers, which include germ cell cancer, sex-cord stromal cancer, small cell carcinoma, and ovarian sarcoma, merely account for less than 5% of OC while the proportion of EOC is surprisingly over 95% [5]. In accordance with distinct histological features, EOC can be further divided into 5 subtypes, encompassing high-grade serous, low-grade serous, endometroid, mucinous, and clear cell ovarian cancer, whose preventative approaches, clinical results, and therapeutic responses widely vary [6]. Although the curative ratio is comparatively satisfying for early-stage EOC (stage I/II), 75% of patients got diagnosed at stage III/IV due to the non-obviousness and non-typicalness of its incipient symptoms and the lack of effective early detection techniques [6]. More infelicitously, in terms of the prognosis of patients with advanced-stage EOC, incurable recurrence approximately accounts for 75% [7] and developing therapy resistance is also largely facilitated [8]. Consequently, there is no doubt that EOC has become a colossal menace to females around the whole world, thus making it an exigent necessity to define novel therapeutic targets and explore new effective therapeutic strategies for EOC.

In recent decades, unprecedented attention has been shifted to cell death for its pivotal role in multifarious aspects of both normal developmental and pathophysiological processes. Currently, based on diverse functional and morphological differences, cell death can be basically split into two general groups, namely accidental cell death (ACD) and regulated cell death (RCD) [9]. Unlike ACD, which is touched off by overwhelming and uncontrollable accidental attacks and/or unexpected injuries, the occurrence of RCD depends on particular signals and precise molecular mechanisms, suggesting that RCD can be regulated via pharmacological or/and genetical approaches [10, 11]. Up to now, thanks to the expeditious and remarkable evolution of relevant fundamental research, many types of RCDs and their underlying mechanisms have been elucidated, consisting of apoptosis, necroptosis, pyroptosis, parthanatos, entotic cell death (entosis), netotic cell death (netosis), lysosome-dependent cell death (LDCD), autophagy-dependent cell death (ADCD), alkaliptosis, oxeiptosis, cuproptosis, and particularly ferroptosis [11–13].

Ferroptosis, a biological term initially put forward in 2012, refers to a unique iron-dependent and non-apoptotic form of RCDs [10]. The occurrence of ferroptosis is triggered by the dysregulation of the lipid peroxidation pathway and iron metabolism, in which acute and chronic oxidative stress plays an essential part [14]. In the past ten years, mounting evidence has collectively demonstrated ferroptosis’s significant role in extensive biological and pathological situations, covering anti-viral immunity, aging, and especially cancer suppression [15], thereby making it a glistening and rising star in the fundamental research field of cell biology and basic medicine. Accumulating research findings have unveiled its tremendous and excellent potential in the treatment of various cancers, such as breast cancer [16], colorectal cancer [17], and notably EOC [18].

In the research of ferroptosis and EOC, an exponential development has been achieved, thereby desperately calling for summative insights. Herein, we firstly provide a systemic brief introduction of ferroptosis. And special focus will be placed on constructing a clear conceptual framework of ferroptosis’s emerging and vital role in EOC, followed with a profound discussion of its potential therapeutic implications. Finally, several existing challenges and future research directions are highlighted. We ardently anticipate that this review can innovatively enlighten the development of EOC therapies from a ferroptotic standpoint.

## Ferroptosis: a specific iron-dependent form of regulated cell deaths

### Brief history and definition

While the findings that erastin and RSL3 (two types of ferroptosis inducers) can trigger iron-dependent and non-apoptotic cell death in particular cancer cells have already been reported as early as in 2003 and 2008 [19, 20], the term and specific concept of “ferroptosis” was not proposed until 2012. Stockwell et al., whose lab originally introduced the concept of ferroptosis, have innovatively defined “ferroptosis” as a distinct type of non-apoptotic and iron-dependent cell death that is triggered by lipid peroxidation [15, 21]. And it was in the year 2018, driven by the Nomenclature Committee on Cell Death (NCCD), that ferroptosis got officially recognized as a member of RCD family [10].

### Concise regulatory mechanisms of ferroptosis

**Fig. 1:**
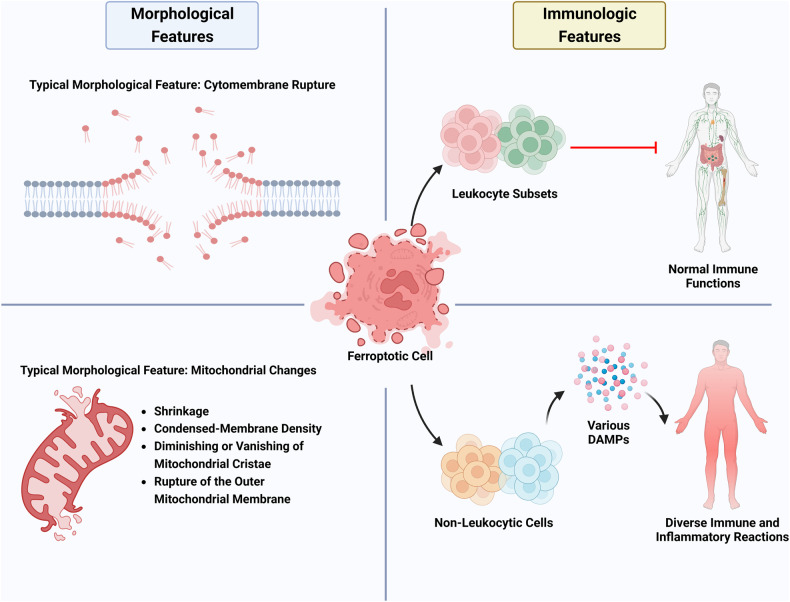
Typical Morphological features of ferroptosis include cytomembrane rupture and several mitochondrial changes (Left). Ferroptosis’s immunologic features are embodied in both leukocytes and non-leukocytic cells (Right). Ferroptotic leukocyte subsets lead to the loss of normal immune functions. Yet in non-leukocytic cells, ferroptosis results in the release of various Damage-Associated Molecular Patterns (DAMPs), thus causing diverse immune and inflammatory reactions.

**Fig. 2:**
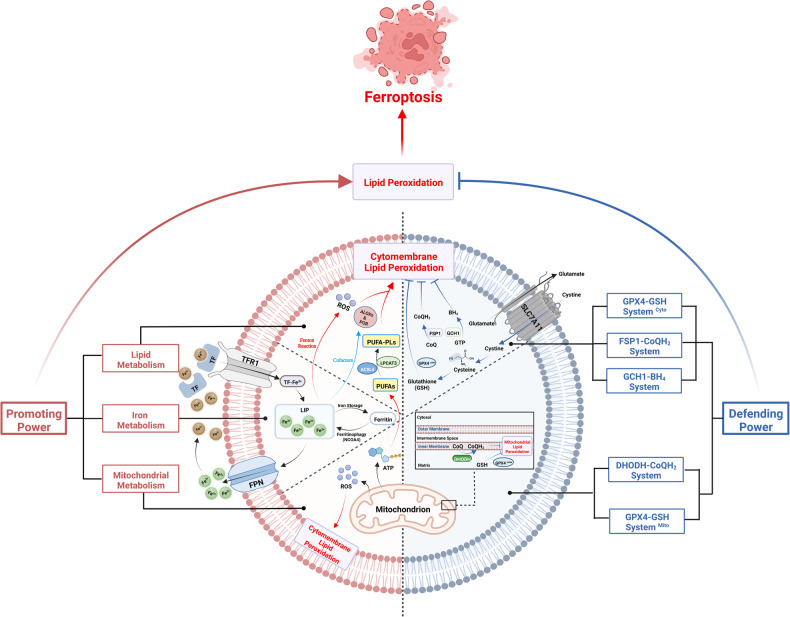
The occurrence of ferroptosis depends on the antagonism between the promoting power (Left) and defending power (right). Only when the promoting power markedly outweigh the defending power can lipid peroxides be excessively accumulated and then trigger ferroptosis.

As a special type of RCD, ferroptosis possesses specific features, especially at morphological and immune levels (Fig. 1). And it also possesses a particular regulatory mechanism of its occurrence. The most typical characteristic of ferroptosis is the lethally excessive accumulation of lipid peroxides, which fundamentally depends on the antagonism between their generation and elimination (Fig. 2) [22]. On the one hand, for ferroptosis-promoting power that boosts the generation and accumulation of lipid peroxides, there are mainly three resources: lipid metabolism, iron metabolism, and mitochondrial metabolism. On the other side, for ferroptosis-defending mechanisms that plays a suppressive role, mainly comprise four cellular antioxidant systems: the GPX4-GSH system, the FSP1-CoQH_2_ system, the DHODH-CoQH_2_ system, and the GCH1-BH_4_ system [22]. Generally, when the power of ferroptosis-promoting cellular activities markedly outweighs ferroptosis-defending capabilities, cytomembranes tend to rupture upon the sustained stimulation of superfluous lipid peroxides and then, ferroptotic cell death can eventually take place [22, 23].

## The emerging and vital role of ferroptosis in EOC: a burgeoning target with extraordinary therapeutic potential

**Fig. 3:**
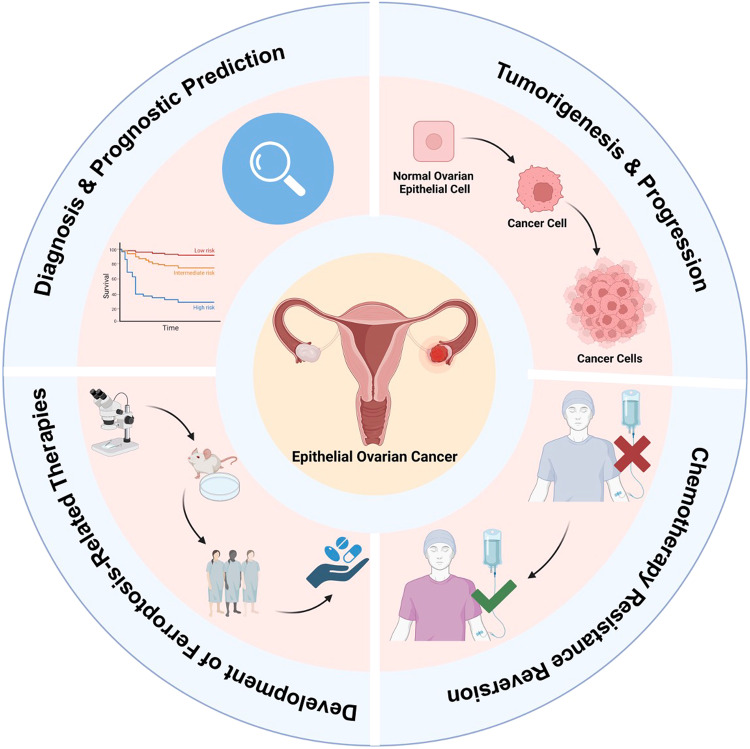
Ferroptosis’s Vital Roles in EOC.

Owing to the mutual struggle of investigators in cancer research and cell biology, numerous findings uncovering ferroptosis’s significant role in tumorigenesis, tumor progression, and antineoplastic therapy of many cancers have been put forward. In this section, our main concentration will be put on the specific association between ferroptosis and EOC. Based on the thorough retrieval and careful reading of relevant literature, we conceptualize the role of ferroptosis in EOC as the following five aspect: its participation in tumorigenesis and progression of EOC, its capability of chemotherapy resistance reversion, development of related therapeutic strategies, prognostic prediction, and ferroptosis resistance (Fig. 3), which are concretely discussed as below.

### Ferroptosis in tumorigenesis and progression of EOC

Even though EOC is the most lethal gynecological cancer and numerous studies have been conducted to explore its pathogenesis and progression, specific mechanisms are still partly unclear. From a ferroptosis-associated perspective, p53 mutations and “iron addiction” can provide possible underlying explanations for this annoying question.

It is well known that mutations of p53 matter a lot in the uncontrollable initiation and progression of various human cancers, including HGSOC. Surprisingly, almost 96% of HGSOC surprisingly exhibit p53 mutation [24]. Commonly, as a remarkable tumor suppressor, p53 is actually a transcription factor able to regulate the synthesis of different RNAs positively or negatively. For example, it can hold up cancer progression via binding to the promotor of the gene encoding SLC7A11, restraining SLC7A11 expression and boosting ferroptosis. In this case, conversely, the mutation of p53 can simply inhibit ferroptosis and accelerate tumor progression. However, in some cancers whose p53 mutations are not that common, it has also been found that normal p53 can suppress ferroptosis through modulating various pathways. For instance, for more than 80% of ovarian clear cell carcinoma (OCCC), a particular subtype of EOC, p53 remains wild type, which correlates with a poorer prognosis and advanced drug resistance, promoted metastasis, and recurrence [25, 26]. The latest research confirmed that the overexpression of MAX3A, a protein with a RING finger domain and an RNA-binding domain, can restrain ferroptosis and boost tumorigenesis through mediating p53 protein degradation [27]. But in human colorectal cancer cells, p53 limits ferroptosis by directly binding to the dipeptidyl peptidase DPP4 to harness lipid peroxidation mediated by NOX [28]. So conclusively, for different subtypes of EOC, the mutation of p53 plays distinct roles and can all possibly be potential therapeutic targets.

On the other hand, as proposed earlier, iron metabolism occupies an important position in ferroptosis, which largely depends on iron levels in cells. In terms of different types of cancers, the roles of iron levels enormously vary from each other. For EOC, in contrast with normal ovarian tissue, the tumor initiation cells (TICs) of HGSOC manifested an apparently higher level of transferrin receptor 1 (TFR1, the iron importer) and an obviously lowered level of ferroportin (FPN, the iron efflux pump), eventually resulting in the excessive accumulation of intracellular iron [29]. And via coercively reducing intracellular iron in TICs, the conspicuously suppressed tumor growth and intraperitoneal dissemination of HGSOC were observed, indicating that the proliferation and invasion of HGSOC TICs largely depended on the raised level iron. Such strong dependence on iron in TICs is called “Iron Addiction”. Owing to the fact that HGSOC TICs particularly rely on the intracellular iron, these cells have been proved more sensitive to ferroptosis inducers. So, it can be an effective option to apply ferroptosis inducers to restrain HGSOC tumor growth and metastases by boosting ferroptotic cell death. This can likely be a novel direction for drug development and clinical intervention. However, in lung cancer, since the defulfurase NFS1 is overexpressed and has been found necessary for tumor progression, the intracellular labile iron level is limited, consequently protecting cancer cells from ferroptosis [30]. Coincidentally, breast cancer cells can lower iron level and facilitate ferroptosis evasion as well by enhancing the expression of promonin 2, a molecule able to discharge iron out of cells through forming ferritin-containing multivesicular bodies [31].

### Chemotherapy resistance reversion

Despite the outstanding improvement achieved in both diagnosis and treatment over the past few decades, the deficiency of effectual early detection techniques and the extremely common establishment of acquired resistance to cisplatin- or carboplatin-based chemotherapy collectively render the survival rate of EOC quite unsatisfactory [7]. For women diagnosed with HGSOC, the overall 5-year survival rate is lower than 40% because 15–25% of patients undergo primary resistance and most remaining patients will develop resistance later [8, 32]. At present, chemotherapy resistance has become a massive hurdle for the clinical management of EOC. In recent years, several research findings have proposed that the use of ferroptosis inducers can open new avenues for tackling this gordian knot.

Previous evidence suggests that the overexpression of ABCB1 (P-glycoprotein/MDR1) is the primary drug transporter leading to multidrug resistance (MDR) in OC treated with taxane drugs and paclitaxel 12–15 [33–35]. In 2019, having applied erastin (a type of ferroptosis inducers) and docetaxel to A2780/Taxol OC cells with upregulated ABCB1, Hai-Hoing Zhou et al. found that erastin could enhance the level of intracellular ABCB1 substrate by suppressing the drug-efflux activity of ABCB1 and subsequently reversed ABCB1-mediated docetaxel resistance in OC [36]. In addition to ABCB1, the presence of persister cancer cells (PCCs) is another relevant mechanism of MDR [37]. Also in 2019, Yanjun Zhao’s team reported the utilization of triggered ferroptotic polymer micelles encapsulating RSL3 (one type of ferroptosis inducers targeting GPX4) could effectively remove drug tolerant PCCs and lower the biomarkers of MDR in the model of human ovarian adenocarcinoma cells both in vivo and in vitro [38]. Furthermore, in 2021, Yinu Wang et al. issued that platinum-tolerant cells and tumors presented the partial expression of cancer stem cells, the Wnt receptor *Frzzled-7* (FZD7) [39]. Deeper exploration confirmed that the overexpression of FZD7 could activate the oncogenic factor *TP63* through Wnt/β-catenin signaling pathway, following with the upregulation of glutathione metabolism pathways, especially GPX4. As mentioned before, GPX4-GSH system is one primary composition of ferroptosis-defending power. GPX4 can transform lethal lipid hydroperoxides (L-OOH) into non-toxic lipid alcohols (L-OH), coupled with suppressing the generation and accumulation of ROS, which can protect cells from ferroptosis [40–42]. Consequently, the elevated expression of GPX4 could protect these PCCs from being harmed by chemotherapy-induced oxidative stress and refraining the occurrence of ferroptosis. In this way, theoretically, treating these PCCs with GPX4 inhibitors (another group of ferroptosis inducers) can promisingly rebuild the sensitivity to platinum-based chemotherapy, which is another vulnerability that can be targeted by contrived therapeutic strategies.

### Research and development of ferroptosis-associated therapeutic strategies

In addition to the function of tumorigenesis, cancer progression, and reversing chemotherapy resistance, another research hotspot of ferroptosis’s role in EOC lies in the development of ferroptosis-associated therapeutic strategies. Here, we sum up the existing research results as 5 categories: broadening the use of existing drugs, combined pharmacotherapy, development of novel nanomaterials, application of small molecule compounds, and defining new ferroptosis-linked therapeutic targets through bioinformatic techniques.

### Broadening the use of existing drugs

The course of developing brand-new drugs that can be effectually used in clinical practices has always been considered as a lengthy process filled with obstacles and difficulties. So, under such conditions, some researchers have turned their attention to many conventional drugs, trying to explore their further utilizations to tackle EOC. And ferroptosis here explains the underlying mechanism of these drugs’ anti-EOC effect. The first successful example is artesunate (ART), one sort of traditional and well-tolerated anti-malarial drug [43]. Researchers here found that not only could ART suppress the in vitro growth of EOC cells, but it could inhibit tumor growth in mice model as well. In terms of the fundamental mechanism, they further proved that exposing EOC cells to high concentration of ART could cause ferroptosis. That was attributed to the generation of ROS, which could directly facilitate lipid peroxidation. In addition, except for anti-malaria drugs, menin-mixed-lineage leukemia (MLL) inhibitors used to treat MLL-rearranged leukemia were also reported able to exert an excellent cytotoxic effect on EOC cells via inducing ferroptosis [44]. Another intriguing instance is about one type of extensively used anesthetics lidocaine [45]. Having been treated with lidocaine, the tested EOC cells exhibited an accumulation of Fe^2+^, iron and lipid reactive oxygen species (ROS), accompanied with attenuated proliferation, invasion, and migration. Subsequent mechanistic experiments confirmed that lidocaine could downregulate the expression of SLC7A11 via enhancing microRNA-382-5p, thereby promoting ferroptosis. The last yet the most outstanding pieces of research evidence here are related to PARP inhibitors (PARPi), one of the first-line drugs primarily applied in BRCA mutant OC [18]. Previous notion considers that PARPi exerts an anticancer function through the basic mechanism called synthetic lethality [46]. However, this study suggested that, instead of synthetic lethality, ferroptosis could also partly account for the antineoplastic function of PARPi Olaparib. Regarding the underlying mechanism, Ting Hong et al. disclosed that the expression of SLC7A11 was downregulated in a p53-dependent way by pharmacological suppression or genetic knockdown of PARP, which subsequently boosted lipid peroxidation and ferroptosis. The far-reaching significance of this study lies in the revealing of a previously unheeded mechanism of PARPi, which indicates that for EOC patients carrying wild-type BRCA1/2, PARPi may be a superb option as well.

### Combined pharmacotherapy

Several studies discovered that in contrast with normal ovarian tissue, some molecules were evidently upregulated in EOC tissue, playing an important part in the advancement of EOC through protecting cancer cells from ferroptosis. As a result, via the combinative use of the inhibitors of these upregulated molecules, ferroptosis inducers or chemotherapy agents, a remarkable anticancer therapeutic effect can be realized. According to Lia Tesfay and colleagues, stearoyl-CoA desaturase 1 (SCD1, an enzyme catalyzing the rate-limiting step in monosaturated fatty acids oxidation) was overexpressed in EOC tissue, which defended cells against ferroptosis by increasing CoQ10, an endogenous antioxidant located in cytomembrane [47]. Conversely, inhibition of SCD1 could concurrently induce cell ferroptosis and apoptosis. Further exploration proved that the anti-cancer capability of ferroptosis inducers could be potentiated by SCD1 inhibitors both in ovarian cancer cell lines and mouse models with orthotopic xenograft. Therefore, combining SCD1 inhibitors with ferroptosis inducers can be a promising strategy for patients with EOC. Apart from SCD1, another fatty acid desaturase was reported abnormally upregulated in ascites-derived OC cells, namely acyl-CoA 6-desaturase (FADS2) [48]. In this study, researchers proved that both SCD1 and FADS2 were related to the increase of intracellular unsaturated fatty acids and enhanced aggressiveness of tested cells. Following experiments showed that the restraining of SCD1/FADS2 could disrupt the cellular/mitochondrial redox equilibrium via directly targeting GPX4 and GSH/GSSG ratio, eventually leading to lipid peroxidation, mitochondrial dysfunction, and eventually ferroptotic cell deaths. These discoveries implicate that it is a viable choice to combine SCD1/FADS2 inhibitors with cisplatin, which can achieve a synergistic effect in harnessing the dissemination of EOC. Moreover, *GALNT14* has also been reported as an evidently upregulated molecule in EOC cells [49]. Based on Hua-Wen li et al., downregulating *GALNT14* could repress both ferroptosis and apoptosis in EOC cells via the EGFR/mTOR pathway. And further assay illustrated that a combinative treatment of an mTOR inhibitor and cisplatin could synergistically boost cancer cell death, hence offering another promising approach for EOC treatment.

### Development of novel nanodrugs

In recent years, with the sustained and rapid prosperity of nanotechnology, numerous nanoparticles and nanomaterials have been developed and utilized in targeted therapy against malignancies for their outstanding targeting ability, high specificity, and minimized shielding effect [50]. In the research area of EOC, as mentioned earlier, in 2019, Yanjun Zhao et al. designed triggered ferroptotic polymer micelles, which simultaneously carried RSL3 [38]. Made of arachidonic acid-conjugated amphiphilic copolymer, the micelles could be triggered by free radical and swiftly release loading drugs in the tumor microenvironment, exerting remarkable cell killing and MDR reversion effects. Another commendable instance is superparamagnetic iron oxide nanoparticles (SPIONs) with excellent stability and convenient modification. It is a group of famous carriers for both nucleic acids and drugs, was of ferroptosis-induction ability in EOC stem cells through causing oxidative stress and lowering autophagy activity, thus collectively retarding cell proliferation, invasion, and tumorigenesis [51]. Using the same superparamagnetic iron oxides (SPIO), Yunhan Zhang et al. chose to incubate SPIO with human serum and confirmed that SPIO-Serum could downregulate GPX4 and xCT to promote lipid peroxidation and ROS generation, which ultimately leaded to ferroptotic cell death in EOC cells in a p53-dependent manner [52].

### Application of small molecule compounds

Apart from nanomaterials and nanoparticles, a crowd of researchers set their sights on small molecule compounds, profoundly tapping their anticancer capacity. Herein, several natural and chemical small molecule compounds have been found able to exert an anticancer efficacy in EOC.

Natural small molecule compounds chiefly encompass a bioactive protein named MAP30, which was subtracted from Momordica charantia (also known as bitter melon or bitter gourd), agrimonolide isolated from Agrimonia Pilosa Ledeb, and carboxymethylated pachyman (CMP) [53–55]. In terms of MAP30, it could exert antineoplastic effect through various mechanisms, mainly including activating AMP-activated protein kinase (AMPK) signaling through CaMKKβ, inducing arrest of cell cycle, regulating cell metabolism of both glucose and lipid in tumorigenesis and progression, and notably triggering ROS-mediated apoptosis and ferroptosis by lifting the intracellular Ca^2+^ level [53]. Besides, it is worth noting that as a natural subtraction from bitter melon, a common edible fruit or vegetable, MAP30 also exhibited superior safety in mice models, further expanding its therapeutic potency. Comparably, targeting SCD1 and subsequently downregulating SLC7A11 and GPX4, arimonolide could touch off both apoptosis and ferroptosis via enhancing the intracellular level of iron, ROS and Fe^2+^ [54]. As for CMP, Tiantian Jing et al. corroborated that it could conspicuously inhibit NRF1/HO-1 signaling pathway and downregulate xCT and GPX4, hereby inducing ferroptotic cell death in EOC cells [55].

The newly defined chemical small molecule compound that possesses antitumor effect in EOC is sodium molybdate [56]. This type of soluble molybdenum (Mo) compound was reported capable of promoting the labile iron pool (LIP), downregulating genes involved in extracellular matrix organization, boosting GSH depletion via the regulation of nitric oxide (NO) generation, which jointly gave rise to ferroptosis and apoptosis. In a brief summary, these small molecule compounds can all be promising and potential therapeutic candidates for EOC patients.

### New ferroptosis-related intervention targets

Last but not least, a cluster of novel therapeutic targets concerned with ferroptosis in EOC have been uncovered as well. The discoveries and defining of these ferroptosis-linked targets ignite the fire of hope of establishing new orientations for EOC intervention. A small class of these newly defined targets are associated with molecules crucial to the occurrence mechanism of ferroptosis, such as ACSL4, GPX4, cysteine, and SLC7A11. As reported by Lin-Lin Ma et al., ACSL4 was significantly upregulated in EOC tissues, which predicted unsatisfactory prognosis yet enhanced cell sensitivity to eratin- and RSL3-triggered ferroptosis [57]. And directly binding to ACSL’s 3’-UTR, miR-424-5p was capable of repressing ACSL4 and deterring ferroptosis, thereby making it an underlying target. Furthermore, Dingxi Li et al. considered GPX4 and intracellular iron levels in EOC cells as a favorable target [58] while Wisna Novera et al. proposed that the intracellular content of cysteine could be another one [59]. Also, as the glutamate/cystine antiporter, SLC7A11 (solute carrier family 7 member 11) plays an elementary role in ferroptosis, targeting which can largely influence the occurrence of ferroptosis [60]. In this March, it has been put forward that long non-coding RNA (lncRNA) ADAMTS9-AS1 inhibited ferroptosis in EOC cells via targeting miR-587/SLC7A11 [61]. Coincidentally, SNAI2 (also known as Slug), which can encode a zinc-finger protein of the SNAI family of transcription factors [62], could also regulate ferroptosis in EOC cells by directly binding to SLC7A11’s promotor [63] in this March as well. Other potential therapeutic targets cover Yes-associated protein 1 (YAP), the unique paralog of a Hippo pathway effector called TAZ [64]. To be more precise, S-phase kinase-associated protein 2 (SKP2), an E3 ubiquitin ligase, was verified as a direct target of YAP. Through upregulating SKP2, YAP was able to indirectly boost ferroptosis in EOC cells, creating itself as a novel therapeutic target for EOC. Moreover, according to the latest research findings published in this June, centrosome and spindle pole-associated protein (*CSPP1*), a centrosome and microtubule-binding protein, was in connection with ferroptosis and tumor microenvironment in EOC cells as well, whose aberrant expression could be regarded as a biomarker for cancer diagnosis [65]. Conclusively, targeting CSPP1 is of special therapeutic value for EOC therapy.

### Prognostic prediction

Nowadays, largely benefited from the brilliant advances of modern biotechnology and information technology, bioinformatic analyses have garnered considerable interests from many researchers in the field of ferroptosis and EOC (Table 1). In 2019, using proteomic, metabolomic, and bioenergetic analyses, Gentric et al. unanticipatedly discovered a metabolic heterogeneity in HGSOC [66], according to which HGSOC patients could be divided into two subgroups: low-OXPHOS (oxidative phosphorylation) and high-OXPHOS. Further experiments revealed that high-OXPHOS tumors exhibited features associated with chronic oxidative stress and ferroptosis, and high-OXPHOS metabolism was significantly related to a better prognosis, suggesting that this metabolic heterogeneity of HGSOC may help predicate the prognostic condition of patients [66]. Later in 2020, based on statistics mainly obtained from two authoritative databases called TCGA (The Cancer Genome Atlas; https://portal.gdc.cancer.gov/) and GEO (Gene Expression Omnibus; https://www.ncbi.nlm.nih.gov/geo/), Huan Wang et al. successfully defined a novel prognostic signature of EOC, encompassing three ferroptosis-related genes (FRGs): HIC1, LPCAT3, and DUOX1 [67]. Moreover, on the basis of these three FRGs, a risk score model able to separate patients into high- and low-risk groups was established and validated. Following functional analysis suggested that in the high-risk group, immune response and immune-related pathways were better enriched, coupled with a microenvironment made of more M2 Macrophage infiltration and higher expression of core immune checkpoint molecules, indicating a worse prognosis. In addition to FRGs, ferroptosis-related lncRNAs (FRLs) have the same potential in EOC as well. In an article published in this January, authors constructed an FRL-signature with nine FRLs, which could be applied in patients’ risk stratification and prognosis prediction [68]. Examples like this have been emerging and can be easily enumerated, yet these mentioned here just suffice to present the remarkable potential of the bioinformatic analyses in EOC prognosis prediction. Coincidentally, as summarized by Jianfa Wu et al., numerous ferroptosis-associated genes have been discovered to be able to influence the staging, recurrence, and survival in endometrial cancer, such as HMOX1, p53, SLC7A11, SAT1, CDKN1A, KEAP1, and NRF2 [69], which are partly different from EOC. The expression level of these genes is also capable of predicting the prognosis of endometrial cancer.

**Table 1:** Ferroptosis-related LncRNAs, genes, and mRNAs with diagnostic and/or prognostic value.

FRGs/FRLs/FRMs	Application value	Reference
**FRLs**: AC007848.1, AC010336.5, AL157871.2, AP001033.1, AC009403.1, AC068792.1, AC011445.1, AC093895.1, LINC01857, LINC00239, AL513550.1	Prognostic Prediction	[78]
**FRLs**: AC138904.1, AP005205.2, AC007114.1, LINC00665, UBXN10-AS1, AX083880.1, AC083880.1, LINC01558, AL023583.1	Prognostic Prediction	[79]
**FRLs**: USP30-AS1, SNHG10, RP5-1120P11.1, RP3-512B11.3, RP11-872J21.3, RP1-313I6.12, RP1-223E5.4, CTC-246B18.8, AC133644.2	Prognostic Prediction	[68]
**FRLs**: RP11-443B7.3, RP5-1028K7.2, TRAM2-AS1, AC073283.4, RP11-486G15.2, RP11-95H3.1, RP11-958F21.1, AC006129.1	Prognostic Prediction	[80]
**FRGs**: DNAJB6, RB1, VIMP/SELENOS, BACH1, STEAP3, ALOX12	Diagnosis & Prognostic Prediction	[81]
**FRGs**: HIC1, LPCAT3, DUOX1	Diagnosis & Prognostic Prediction	[67]
**FRGs**: NFS1, ATG7, G6PD, VDAC2, SLC3A2, MAP1LC3C, ACSL3, PTGS2	Prognostic Prediction	[82]
**FRGs**: LPCAT3, ACSL3, CRYAB, PTGS2, ALOX12, HSBP1, SLC1A5, SLC7A11, ZEB1	Prognostic Prediction	[83]
**FRGs**: SLC7A11, RB1, LPCAT3, GCH1, PCBP2, STEAP3, ZFP36	Diagnosis & Prognostic Prediction	[84]
**FRGs**: ALOX12, ACACA, SLC7A11, FTH1, CD44	Prognostic Prediction	[85]
**FRG:** OGN	Prognostic Prediction	[86]
**FRMs**: CDKN1B, FAS, FOS, FOXO1, GABARAPL1, HDAC1, NFKB1, PEX3, PPP1R15A, SIRT2, IFNG, IL24, MTMR14, RB1	Prognostic Prediction	[87]

### Ferroptosis resistance in EOC

While inducing ferroptosis can be a superb novel therapeutic strategy in the fierce battle against EOC, amassing evidence suggests that EOC cells can gradually develop resistance to ferroptosis through a variety of mechanisms. As pointed out by Liu et al., ferroptosis resistance could be established in EOC cells through a drawn-out exposure in erastin [70]. Sustainably activating the reverse trans-sulfuration pathway, these cells would be accustomed to cystine deprivation by constitutively activating NRF2 and subsequently upregulating cystathionine β-synthase (CBS), the key enzyme for cysteine biosynthesis, which contributed to the reduction of their sensitivity to eratin-induced ferroptosis. Likewise, confronting the same problem, Anna Marina Battaglia et al*.* found that in EOC cells, the sensitivity to erastin largely relied on the intracellular free iron level [71]. By extra using iron chelators, the free iron level in cells could be lowered and erastin-induced ferroptosis would be counteracted, bringing out ferroptosis resistance. However, the sensitivity to ferroptosis could be reestablished though the combinative use of low-dose ferlixit and erastin, laying a foundation for such combined therapy in future clinical use. Besides, another study raised that as a sensor of cell density and the predominant effector of the Hippo pathway in tested EOC cells, transcriptional coactivator with the expression of PDZ-binding motif (TAZ) is one determinant factor in regulating ferroptosis sensitivity and resistance. Specifically, enhanced expression of TAZ directly targeted gene ANGPTL4, thus improving cells sensitivity to ferroptosis through the activation of NOX2 [72]. Furthermore, equipped with advanced techniques such as genome-wide CRISPR-Cas9 suppressor screens and lipidomic profiling, Yilong Zou and colleagues firstly identified peroxisomes, the oxidative organelles, as crucial contributors to determine ferroptosis sensitivity in human renal and ovarian carcinoma cells [73]. Accordingly, the downregulation of peroxisomes could suppress ferroptosis through restraining the synthesis of polyunsaturated ether phospholipids (PUFA-ePLs), the substrate of lipid peroxidation, hence transforming the EOC cells from a ferroptosis-sensitive status to resistant. So, we may re-sensitize the ferroptosis-resistant EOC cells via upregulating the peroxisome-ether phospholipid axis. With the progressive prevalence of widespread chemotherapy or drug resistance, deeper exploration of the mechanisms associated with ferroptosis resistance in EOC is irrefutably indispensable. Only when we completely elucidate its underlying mechanisms can we ultimately figure out effective intervention means to overcome this intractable trouble and benefit more EOC patients.

## Conclusion and future directions

Conclusively speaking, with distinct yet complicated promoting and defending mechanisms, ferroptosis is an iron-dependent, non-apoptotic form of regulated cell deaths driven by lipid peroxidation, which has gained unparalleled concern in recent years for its pivotal role in numerous pathological contexts. In the research field of EOC, ferroptosis has remarkably stood out as a burgeoning intervention target by virtue of its spectacular therapeutic potential. In addition to its involvement in the tumorigenesis and progression of EOC, it can miraculously reverse chemotherapy resistance as well. Indisputably, the development of ferroptosis-associated therapeutic strategies can provide more choices of drugs and therapies for EOC patients, coupled with a tangible effective prediction of prognosis, all of which collectively offering patients with a brand-new and amazingly promising treatment landscape.

Nevertheless, despite the myriad, marvelous breakthroughs having been made in the research area of ferroptosis and EOC, the answers to many additional mysteries are still cloaked in heavy fog, waiting to be further explored and eventually unveiled. For example, as mentioned earlier, OC is a disease of extremely high heterogeneity on histopathological level. In addition to EOC, OC can be further classified into several different subtypes, such as sex-cord stromal cancer, germ cell cancer, ovarian sarcoma, and small cell carcinoma. And even EOC consists of several subtypes. However, according to comprehensive and careful bibliographical retrieval and studying, we find that the overwhelming majority of existing research findings were restricted to EOC, especially HGSOC, which might be due to its high prevalence. The differences of ferroptosis’s function between these subtypes of OCs and EOC is still unclear. Whether ferroptosis plays a similar role and if ferroptosis has the analogous therapeutic potential in other types of OCs definitely need more investigation. Besides, how can we accurately examine and confirm the subtypes sensitive to ferroptosis-associated therapy in clinical practice? And while emerging evidence has proved that a combination of ferroptosis-related therapeutic strategies and the existing treating methods, such as the ionization radiation [74], immunotherapy [75], and chemotherapy [36], can possibly realize a synergistic efficacy and achieve a better anticancer effect, how can we effectively select the optimal combination for EOC patients diagnosed at different stages of various subtypes? Moreover, as for the prognosis-predictive models or systems whose establishment largely relies on bioinformatic analyses, it is not clear how to evaluate their accuracy under real clinical circumstances either. Also, it has been unveiled that the chemotherapy resistance of endometrial cancer is relevant to ferroptosis [76]. For Cisplatin and Paclitaxel, endometrial cancer patients with a lower score of ferroptosis commonly exhibited a higher IC50, suggesting that chemotherapy resistance is more possible to be developed in these women [77]. Does this happen to EOC patients as well? How can we find an effective solution to avoid this problem and treat patients under such condition? Apparently, all of these above-mentioned problems are calling for the conduction of more clinical trials on a larger scale. Apart from this, with an aim of successfully conquering chemotherapy resistance, one of the awfully thorniest problems in EOC treatment, the elucidation of its underlying connection with ferroptosis resistance may offer us new insights. More fundamentally, although there are many types of ferroptosis inducers capable of suppressing tumor growth via triggering ferroptosis in vitro, how can we safely and efficiently induce ferroptotic cell death merely in EOC cells without harming peripheral normal cells in vivo still remains unknown, making additional studies an absolute must.

Hopefully, we envision that the following few years can witness the emergence of more remarkable research results related to ferroptosis and EOC. And it is our unswerving belief that with mutual efforts, ferroptosis, a burgeoning target with extraordinary therapeutic prospects, will be effectively and widely employed in the treatment of EOC.
